# Dark Anaerobic Conditions Induce a Fast Induction of the Xanthophyll Cycle in *Chlamydomonas reinhardtii* When Exposed to High Light

**DOI:** 10.3390/microorganisms12112264

**Published:** 2024-11-08

**Authors:** Cecilia Faraloni, Eleftherios Touloupakis, Giuseppe Torzillo

**Affiliations:** 1 Istituto per la Bioeconomia, Consiglio Nazionale delle Ricerche, Via Madonna del Piano 10, Sesto Fiorentino, 50019 Firenze, Italy; giuseppe.torzillo@cnr.it; 2 Istituto di Ricerca sugli Ecosistemi Terrestri, Consiglio Nazionale delle Ricerche, Via Madonna del Piano 10, Sesto Fiorentino, 50019 Firenze, Italy; eleftherios.touloupakis@cnr.it; 3 Centro de Investigación en Ciencias del Mar y Limnologίa, Universidad de Costa Rica, San Pedro, San José 2060, Costa Rica

**Keywords:** photosynthesis, state transition, fluorescence, xanthophyll cycle, photoinhibition, anaerobiosis, plastoquinone, redox conditions

## Abstract

Background: Dark anaerobiosis promotes the acidification of the thylakoid lumen and a reduction in the plastoquinone (PQ) pool. The relationship between the reduction in the PQ pool in the dark and the induction of the xanthophyll cycle under high light stress was investigated in *Chlamydomonas reinhardtii*. Methods: To achieve a comprehensive oxidative/reductive (aerobic/anaerobic conditions) state of the PQ pool, cultures were bubbled with air or nitrogen for 4 h. To induce the xanthophyll cycle, the cultures were then irradiated with 1200 µmol_photons_ m^−2^ s^−1^ white light for 1 h. Results: The anaerobic cultures exhibited a stronger induction of the xanthophyll cycle with a 3.4-fold higher de-epoxidation state than the aerobic cultures. Chlorophyll fluorescence measurements showed that this response was influenced by the previous redox state of the PQ pool, and that dark anaerobiosis triggers physiological responses, such as exposure to high light. Thus, the photosynthetic apparatus in anaerobic cultures was already alerted, at the moment of high light exposure, to give an appropriate response to the stress with a stronger induction of the xanthophyll cycle than in aerobic cultures. Conclusions: Our results provide new information on the importance of the redox signaling pathway and highlight the importance of the reductive conditions of the PQ pool in regulating the physiological responses of photosynthetic organisms to stress.

## 1. Introduction

In order to maintain efficient photosynthesis, microalgae must adapt to considerable fluctuations in light intensity. Light-independent regulatory processes, such as dark respiration, contribute to the optimization of the carbon fixation process [1]. It is noteworthy that dark respiration can also lead to a reduction in the plastoquinone (PQ) pool. Evidence for a respiratory electron transport pathway—i.e., chlororespiration involving the redox component of the chloroplast—has been observed in some eukaryotic algae and has been well documented in the microalga *Chlamydomonas reinhardtii* (*C. reinhardtii* hereafter) [2,3,4]. In the dark, chlororespiration causes a reduction in the PQ pool, whereby electrons are transferred from nicotinamide adenine dinucleotide phosphate (NADPH) to the PQ pool. Chlororespiration in *C. reinhardtti* appears to be stimulated when acetate is added to the growth medium, and it has been associated with a decrease in fluorescence yield and an increase in non-photochemical quenching of chlorophyll fluorescence (NPQ) [5,6].

Under high light stress, the dissipation of excess absorbed light energy occurs via non-photochemical quenching of chlorophyll fluorescence—a harmless non-radiative pathway of energy dissipation. These defensive strategies for dissipating excess energy involve the synthesis of xanthophyll cycle pigments, which is promoted by acidification of the thylakoid lumen [7,8,9,10,11]. The xanthophyll cycle consists of the formation of zeaxanthin through the de-epoxidation of violaxanthin via antheraxanthin in an enzymatic reaction catalyzed by a de-epoxidase during exposure to high light; zeaxanthin is converted back to violaxanthin via anthereaxanthin with the help of the enzyme epoxidase [12,13]. PQ reduction has been shown to be involved in the regulation of other photosynthetic processes, including photoprotective responses, by regulating pigment composition [14].

The relationship between the PQ pool reduction degree and the induction of the xanthophyll cycle under light stress has been extensively investigated in both higher plant species and in microalgae [13,15,16,17]. However, the involvement of PQ pool reduction in the stimulation of the xanthophyll cycle by chlororespiration has received much less attention [18,19]. The results reported in this work show that *C. reinhardtii* cultures incubated under prolonged dark anaerobic conditions (e.g., state 2 cultures) exhibit greater induction of the xanthophyll cycle than the dark aerobic cultures (e.g., state 1 cultures) when subsequently exposed to high light. Chlorophyll fluorescence measurements showed that the response of the xanthophyll cycle when submitted to high light was influenced by the previous redox state of the PQ pool. Previous experiments have shown that anaerobiosis can increase the induction of the xanthophyll cycle even in low light and during hydrogen production in *C. reinhardtii* [20]. The cyanobacterium *Synechocystis* was found to accumulate photoprotective carotenoids, xanthophylls, echinenone, and β-carotene, which were detected in outdoor low-density cultures, which were also used to induce hydrogen production [21]. These studies demonstrated that different pathways can regulate the adaptation of cells to anoxia and photo-oxidative stress, and the results demonstrate that in hydrogen-producing cultures, the presence of a larger xanthophyll pool and the content of antioxidant carotenoids were important for the photoprotection of PSII during the hydrogen process. Based on these experiments, the effect of anaerobiosis on the induction of the xanthophyll cycle, but under high light, was investigated, in order to test if it was possible to obtain a fast synthesis of a higher amount of zeaxanthin than under aerobic conditions.

## 2. Materials and Methods

### 2.1. Strain and Culture Conditions

The *C. reinhardtii* wild type was kindly provided by Prof. Udo Johanningmeier from the University of Halle in Germany. Axenic cultures were grown in 0.5 L Pyrex-glass tubes at 28 °C in TAP medium [22] under continuous white light of 60 µmol_photons_ m^−2^ s^−1^. For the experiments, the cultures were diluted to a chlorophyll concentration of 15 mg L^−1^ during the exponential growth phase. Anaerobiosis was induced by dark incubation of the cultures and by bubbling in pure nitrogen (anaerobic conditions) at a rate of 860 mL min^−1^. Aerobiosis was maintained in the dark by bubbling the cultures with filtered compressed air [23,24,25]. To achieve comprehensive oxidative/reductive (aerobic/anaerobic) conditions of the PQ pool, the cultures were kept under these conditions for 4 h, i.e., until the time when no further changes in photosynthetic parameters were detected. To induce the xanthophyll cycle, the cultures were then irradiated with 1200 µmol_photons_ m^−2^ s^−1^ of white light for 1 h. During the following high light exposure, nitrogen insufflation was maintained in the anaerobic culture to prolong the permanence of the PQ pool in a reductive state [26]. All experiments were repeated three times.

### 2.2. Analytical Procedures

Chlorophyll a (Chl*a*), chlorophyll b (Chl*b*), and the total carotenoids were determined spectrophotometrically in 90% acetone [27].

On the same extracts, the concentrations of individual carotenoids were obtained by a reversed-phase Beckman System Gold HPLC (module 125 solvent) detector (DAD), model 168 Nouveau, column Luna, C8 (Phenomenex, Torrance, CA, USA), according to [28]. Carotenoids were identified by comparing the retention time and the spectrum with the standards, HPLC-grade (Sigma-Aldrich, Louis, MO, USA). Quantification was obtained using the calibration curve made with the relative standard.

The analysis was performed in triplicate.

### 2.3. Fluorescence Measurements

Chlorophyll *a* fluorescence measurements were performed online and in parallel cultures, using two pulse-amplitude-modulation fluorometers (PAM-2000, H. Walz Effeltrich, Germany). The optical fibers of the fluorometers were placed on the surface of the culture vessels, perpendicular to the direction of the incident light [29]. The nomenclature of fluorescence follows van Kooten and Snel [30]. The minimum fluorescence, F_0_, was measured with modulated light (<0.3 µmol m^−2^ s^−1^) from a light-emitting diode (peak wavelength at 655 nm, 600 Hz) provided by a Schott K2 1500 electronic halogen lamp (model FL-103/E.220, H. Walz) to increase the fluorescence yield to the maximum F_m_ value under dark incubation or (F’_m_) under light exposure. Non-photochemical quenching (NPQ) was calculated as the Stern–Volmer quenching coefficient NPQ = F_m_/F’_m_ − 1 [31]. All measurements were repeated three times. The kinetics of chlorophyll fluorescence induction (OJIP transient) were recorded using an induction fluorometer plant efficiency analyzer (PEA, Hansatech, Norfolk, UK). Fluorescence induction kinetics were recorded within a time span of 50 μs to 1 s with a data acquisition rate of 10^5^ readings s^−1^ for the first 2 ms; thereafter, it was then switched to 10^3^ readings s^−1^ [32,33]. The measurements were performed by pointing the probe directly at the surface of the culture vessels. Each chlorophyll α fluorescence induction curve was analyzed using “BiolyzerHP3” software, version 3.0.30.1. The following parameters were calculated from the fluorescence measurements [32]:

- Minimum fluorescence yield, F_0_, recorded at 50 μs.
- Maximum fluorescence yield, F_m_.
- M_0_ = 4 (F300μs − F_0_)/(F_m_ − F_0_), which corresponded to the net rate of the reaction center closure, where it increases by means of trapping and decreases by means of electron transport.
- The variable fluorescence at phase J, V_J_ = (F_J_ − F_0_)/(F_m_ − F_0_), which is considered to be a good indicator of the plastoquinone pool redox state.
- The maximum quantum yield of photosystem II (PSII) for primary photochemistry, Φ(P_0_), calculated as F_v_/F_m_ = (F_m_ − F_0_)/F_m_.
- The quantum yield of the electron transport ΦE_0_, ΦE_0_ = Φ(P_0_) × Ψ_0_, where Ψ_0_ is the efficiency with which a trapped exciton can move an electron further than Q_A_—(primary quinone electron acceptor) into the electron transport chain.
- The parameter S_m_, a measurement of the energy needed to reduce Q_A_ completely, was calculated by dividing the area by F_v_.

All measurements were repeated three times.

### 2.4. Oxygen Measurements

The photosynthetic oxygen evolution rates of the cultures were measured using a YSI model5300 biological oxygen monitor (Yellow Spring Instrument Co., Inc., Yellow Springs, OH, USA). Oxygen evolution measurements were performed at a saturating irradiance of 850 µmol_photons_ m^−2^ s^−1^ and a constant temperature of 28 °C. The samples were removed from the culture vessels while minimizing turbulence and immediately transferred to the oxygen measurement cuvettes and flushed with nitrogen prior to the measurements. All measurements were repeated three times.

### 2.5. Statistical Analyses

Student’s *t*-test was applied to evaluate the differences in the effect of anaerobiosis on *C. reinhardtii* cultures. Data were analyzed by one-way ANOVA and significant differences among means between data obtained in aerobic and anaerobic cultures (*p* < 0.05) were determined by Tukey’s multiple range test. Statistical analyses were performed using the PRISM 5 software package.

## 3. Results

To test whether keeping the culture under dark anaerobic conditions can induce the xanthophyll cycle, two cultures under different redox conditions were first kept in the dark for 4 h under either aerobiosis or anaerobiosis and then exposed to high light for 1 h. During this time, Chl a fluorescence, photosynthetic activity, and xanthophyll cycle pigment measurements were monitored online.

### 3.1. Changes in Chlorophyll Fluorescence Quenching and Transient

**Figure 1:**
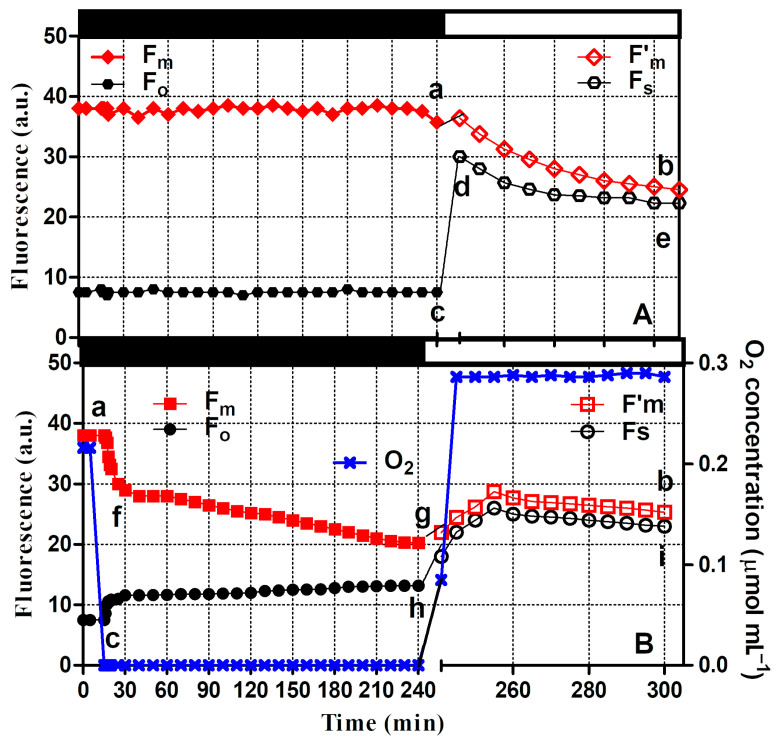
Effect of air (**A**) and nitrogen (**B**) bubbling under dark (closed symbols) and high light exposure (open symbols bar), on basal chlorophyll fluorescence (F_0_ and F_s_, under dark (black bar) and light (white bar) conditions, closed and open black circles, respectively) and maximum chlorophyll fluorescence (F_m_ and F’_m_, under dark and light conditions, closed and open red squares, respectively). The closed and open symbols refer to data measured under dark and light conditions, respectively. The changes in oxygen concentration, in the culture bubbled with nitrogen, are indicated (blue cross). For each parameter, different letters indicate significant differences between the values.

In the culture maintained under aerobiosis, the measurements of chlorophyll fluorescence indicated changes showing that both the minimum and maximum fluorescence yields, F_0_ and F_m_, respectively, remained constant (Figure 1A). In the culture flushed with nitrogen in the dark, F_0_ increased within 15 min of the occurrence of anaerobiosis, as indicated by the O_2_ trace, reflecting the increased reduction in the PQ pool. F_m_ significantly decreased as a consequence of the reduced electron transport efficiency from PSII to PSI, and thereafter, with the permanence of the anaerobic phase, changes in both F_0_ and F_m_ followed the initial trend, although at a lower rate (Figure 1B). During the high light exposure of the aerobic culture, the maximum fluorescence yield, F’_m_, decreased sharply, by more than 50%. The minimum fluorescence in the light, F_s_, increased more than 4-fold within the first minute and decreased to a value 3-fold higher than that of the culture kept in the dark (Figure 1A). In the anaerobic culture, both F_s_ and F’_m_ began to increase with high light exposure by 40% and 50%, respectively, within 15 min and showed a further slight decrease during the subsequent exposure period (Figure 1B).

**Figure 2:**
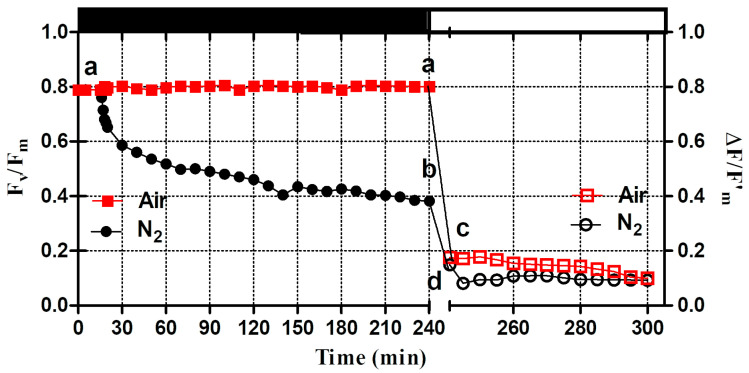
Effect of air (red square) and nitrogen (black circle) bubbling under dark (black bar) and high light exposure (white bar) on the value of maximum PSII quantum yield (F_v_/F_m_) and effective maximum PSII quantum yield (ΔF/F’_m_). Closed and open symbols refer to data measured in dark and light conditions, respectively. Different letters indicate significant differences between the values.

The values of quantum yield of PSII photochemistry (F_v_/F_m_) dropped from 0.789 to 0.680 after 15 min of nitrogen bubbling (Figure 2), which can be attributed to the changes in F_0_ and F_m_ when anaerobiosis occurred. During the further permanence in dark anaerobic conditions, F_v_/F_m_ continued to decrease at an almost constant rate and reached the value of 0.385, after 240 min of dark incubation. The F_v_/F_m_ value in the aerobic culture remained constant and maintained the same initial value during the entire period of permanence in the dark.

Upon subsequent exposure to high light, both the aerobic and anaerobic cultures showed strong changes in fluorescence yields within 15 min after exposure. In the culture kept under anaerobiosis, the effective quantum yield of PSII—i.e., the ΔF/F’_m_ ratio—dropped below 0.100, while in the aerobic culture it dropped to 0.178 (Figure 2). During subsequent exposure to high intense light, the ΔF/F’_m_ ratio remained almost constant in both cultures.

**Figure 3:**
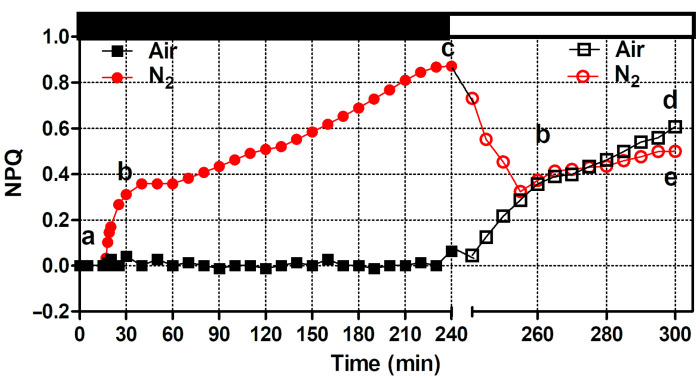
Effect on non-photochemical quenching (NPQ) of air (black squares) and nitrogen (red circles) bubbling under dark (black bar) and high light exposure (white bar). Closed and open symbols refer to data measured in dark and light conditions, respectively. Different letters indicate significant differences between the values.

The changes in the PSII efficiency of the cultures were also reflected in the measurements of NPQ (Figure 3). A sharp increase in NPQ was observed in the nitrogen-bubbled culture after about 15 min of dark incubation. After an initial increase to 0.357, the NPQ reached its maximum value of 1.0 after 240 min of dark incubation. In the culture maintained under aerobic conditions, no increase in NPQ was observed during the time it was kept in the dark (Figure 3). After exposure to high light, an opposite behavior of NPQ was observed within 15 min; it sharply increased to 0.29 in the aerobic culture, while it decreased to 0.50 in the anaerobic culture. During the following 30 min of exposure to high light, both cultures showed an increasing NPQ, following the same trend and showing close values (Figure 3).

**Figure 4:**
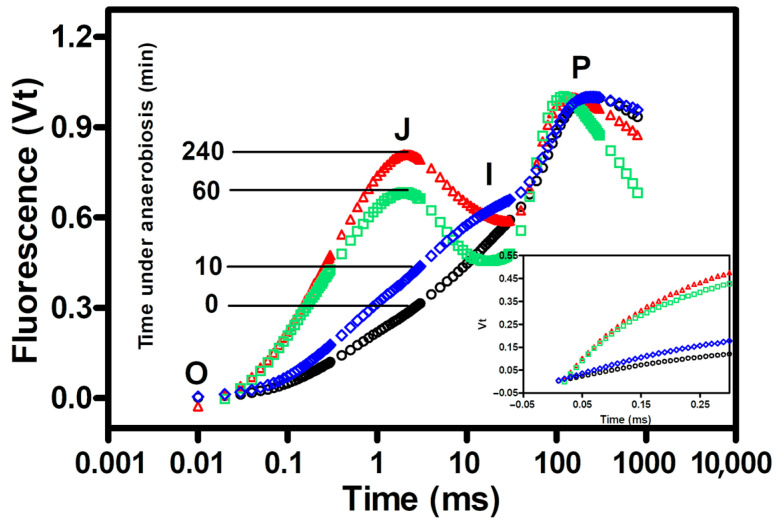
Effect of nitrogen bubbling under dark conditions on the chlorophyll a fluorescence (OJIP) transient: start of the experiments, aerobic conditions (black circles), after 10 min of nitrogen bubbling (blue diamonds), after 60 min of nitrogen bubbling (green squares), and after 240 min of nitrogen bubbling (red triangles). The insert shows the initial fluorescence rise (from 50 ms to 0.6 ms).

Further information on the degree of reduction in the PQ pool was obtained by measuring the chlorophyll fluorescence transient and calculating the JIP test parameters in both the aerobic and anaerobic cultures. Normalized chlorophyll fluorescence data for both F_0_ and F_m_ are shown in Figure 4, to facilitate the comparison between cultures under aerobic conditions and after the occurrence of anaerobiosis. Cultures maintained under aerobic conditions by air bubbling showed no changes in the transient. Figure 4 clearly shows that the anaerobic conditions favored a sharp increase in the J-step and an increase in the initial slope of the fluorescence (insert Figure 4). The fluorescence value in the J-step is considered a good indicator of the level of reduction of Q_A_ and its increase fits well with electron accumulation at the PQ level under anaerobic conditions.

Detailed information on the changes in OJIP transient parameters are reported in Table 1. During the permanence of 240 min of culture under anaerobiosis, the value of the initial slope of the transients, M_0_, increased almost threefold, V_J_ more than doubled compared to the initial value, and ψ_0_ and ΦE_o_ decreased by 53% and 84%, respectively. As expected, no significant changes in the values of any of the parameters tested were observed in aerobic culture (Table 1).

### 3.2. Oxygen Evolution Changes

**Figure 5:**
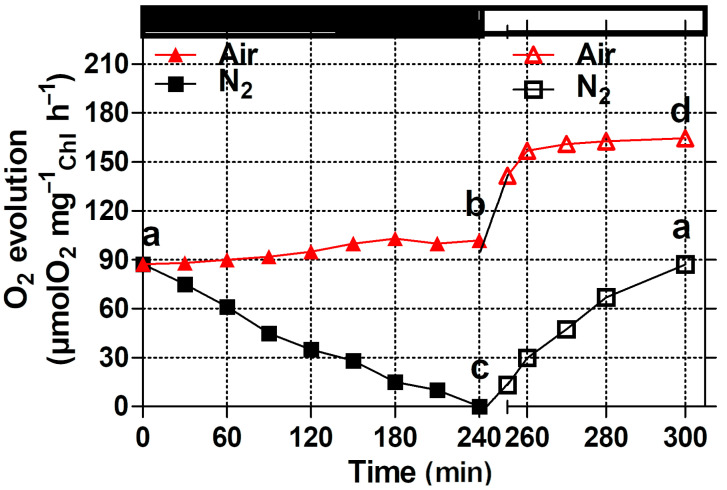
Effect of air (red triangle) and nitrogen (black square) bubbling under dark (black bar) and high light exposure (white bar) on oxygen evolution rate. Closed and open symbols refer to data measured under dark and light conditions, respectively. Different letters indicate significant differences between the values.

The measurements of oxygen evolution during the dark incubation of both anaerobic and aerobic cultures showed differences in their photosynthetic activity (Figure 5). The oxygen evolution rate showed a linear decrease in the anaerobic culture. After 240 min of anaerobiosis, no more light-induced oxygen evolution could be detected, indicating a strong loss of functional PS II centers. In contrast, photosynthetic activity under dark aerobic conditions showed no significant change compared to the initial O_2_ evolution.

Upon subsequent exposure to high light, which was accompanied by a recovery of fluorescence yield, the oxygen evolution in the anaerobic culture increased again and reached the original level after 60 min of exposure, reflecting the recovery of PSII activity and a functional link between the two photosystems. Under aerobic conditions, no significant changes in oxygen evolution were observed during the dark phase, while in the subsequent high light phase, oxygen evolution increased by about 40% of the initial value, most likely due to the culture adapting to the high light conditions.

### 3.3. The Xanthophyll Cycle Under High Light in Anaerobic and Aerobic Cultures

During incubation in the dark, under both anaerobic and aerobic conditions, the composition of the xanthophyll pool showed no significant differences between the two cultures, even if it appeared slightly increased in the anaerobic culture, as shown by the increase in antheraxanthin content, while the content of zeaxanthin was negligible (Table 2). During the subsequent exposure of the two cultures to high light under different redox conditions, the changes in the composition of the xanthophyll cycle pigments became more evident. Within the first 15 min under high light, in the anaerobic culture, the violaxanthin content decreased by 14%, while the antheraxanthin content increased twofold. During the same period, the zeaxanthin content increased more than 2-fold. The xanthophyll cycle was also clearly induced in the aerobic culture, but the change took place to a lesser extent. The corresponding de-epoxidation state (DES) in the anaerobic and aerobic cultures was 0.152 and 0.076, respectively (Table 2). When the cultures were further exposed to high light, the conversion of violaxanthin to zeaxanthin via antheraxanthin increased further in the anaerobic culture. After 60 min of exposure to high light, the DES of xanthophylls increased to 0.514 in the anaerobic culture due to the different conversion rate of violaxanthin in the two cultures, while it only increased to 0.151 in the aerobic culture.

Analysis of the xanthophyll pool showed that the total amounts of violaxanthin, antheraxanthin, and zeaxanthin in both cultures did not change significantly during the experiment, nor did the chlorophyll content (Table 2).

To clearly demonstrate that the enhanced induction of the xanthophyll cycle in the anaerobically incubated culture was related to the preceding reductive conditions achieved during the dark anaerobic incubation, two *Chlamydomonas* cultures, one bubbled with nitrogen and the other one with air, were exposed to high light without prior dark incubation. During the 1 h exposure to high light, both cultures showed the same trend in violaxanthin, antheraxanthin, and zeaxanthin content and the same de-epoxidation state. In addition, no significant differences in fluorescence yield and oxygen evolution rates were observed in the two cultures.

## 4. Discussion

The results reported here show distinctive evidence of a fast xanthophyll cycle induction in previously adapted dark anaerobic cultures of *Chlamydomonas reinhardtii* (Table 1). Anaerobic conditions in the dark caused a strong reduction in the PQ pool, characterized by decreased fluorescence yield, high non-photochemical quenching, and low oxygen evolution rates (Figure 1, Figure 3 and Figure 5).

The reduction state of the PQ pool plays an important role in photoprotective mechanisms, by the regulation of electron transport at many levels, from plastid gene expression to the distribution of light between the two photosystems, e.g., state transition [26,34]. State 2 is traditionally induced by the combination of darkness and anaerobiosis, conditions that disable both mitochondrial respiration and chlororespiration [35,36]. Under these conditions, the PQ pool is effectively reduced by type II NAD(P)H dehydrogenase (NDA2) [37], allowing the transition to State 2 by moving the mobile fraction of the LHCII complex from PSII to PSI and balancing the light excitation energy between PSII and PSI. The state transitions and the reduction in the PQ-pool correlate well with each other when induced by dark incubations without chemical additives.

Recent studies suggest that, in *C. reinhardtii*, a non-photochemical reduction in PQ is more active than in plants, which enables this microalga to react to the changes in light intensity and the PQ pool redox state by different mechanisms [38]. One mechanism is dependent on light intensity and likely reflects the role that algal state transitions have been shown to play in photoprotection [39,40]. On the other hand, other mechanisms react to the PQ pool’s redox state and balance light utilization amongst the photosystems in low light. It is worth noting that in our experiment, the fluorescence yield, as can be seen in the unchanged value of F_0_ and F_m_ in the aerobic culture in the dark, did not show the PQ-reductive effect of chlororespiration, in spite of the presence of acetate. This was due to the fact that vigorous air insufflation created oxidative conditions, which kept the PQ oxidized. The strong reduction in the PQ pool in dark anaerobic cultures was confirmed by the significant increase in the V_J_ parameter under these conditions, while aerobic cultures in the dark showed no changes in this parameter.

The shape of the Kautsky curve after 2 h of dark anaerobic incubation showed a great loss of fluorescence and resembled that observed by Strasser [41]. Indeed, the changes in the shape of the Kautsky curve in the anaerobic cultures were in agreement with those obtained with cultures pre-illuminated under different light strengths, or those detected in cultures with different dark-adaptation times after illumination of the cells with high light, reflecting different levels of filling up of the photosynthetic carriers. The increase in V_J_ was found in leaves of *A. thaliana* maintained under dark anaerobic conditions and it was associated with the induction of state 2 transition, due to the over-reduction of the PQ pool [42]. The results obtained with *C. reinhardtii* were in agreement with these findings, confirmed that PQ pool reduction was promoted by anaerobiosis in the dark (Table 1).

Previous results obtained in dark anaerobic cultures of *C. reinhardtii* showed that the strong increase in NPQ occurring under these conditions was suppressed by the addition of the ionophore nigericin [20]. This demonstrates that the increase in NPQ under dark anaerobiosis is closely linked to the generation of a proton gradient across the thylakoid membranes and the resulting acidification of the thylakoid lumen [5,43]. The kinetics of relaxation during the switch from anaerobic to aerobic conditions corresponded to the recovery from the occurrence of the ΔpH-related NPQ [44]. Otherwise, the focus of the present manuscript was to put in evidence if the anaerobic condition could have a positive effect on the induction of the physiological response to photooxidative stress, like the xanthophyll cycle, also in concomitance of high light intensity, which basically promotes it. The xanthophyll cycle is considered a rapid stimulatory mechanism in response to excess energy activated within minutes by low pH in the lumen and a rapid defensive strategy to protect PSII from high light stress. Moreover, the PsbS protein involved in photoprotective energy dissipation was found to be bound to both chlorophyll and xanthophylls, suggesting that the protonation of PsbS may lead to the quenching of single excited chlorophyll by transferring energy directly from chlorophyll to zeaxanthin [45]. The results showed a higher and faster zeaxantin production under both anaerobiosis and high light intensity than with only high light exposure, suggesting a positive contribution of the previous anaerobic over-reduction of the PQ-pool to the induction of the xanthophyll cycle.

It has already been established that the conversion rate of plastoquinone to plastoquinol (PQ/PQH_2_) can be considered as a biological measurement tool to detect energy imbalances between photochemistry and biochemistry [14,46]. Our results show that the degree of reduction in the PQ pool plays a crucial role in the activation of the xanthophyll cycle in *C. reinhardtii* cultures. In previous experiments, the high PQ pool reduction level, induced by anaerobic dark conditions, was effective for the highest induction of the xanthophyll cycle under low light conditions within 24 h [20]. In the present work, the zeaxanthin content in the anaerobic culture reached a considerable increase (45.52 mmol mol^−1^ Chla) within 1 h, almost four times higher than under aerobic conditions, a shorter period than in the previous study, due to the high light exposure.

The dark anaerobic conditions simulated to some extent a situation of “light stress” due to an excess of reducing power in the anaerobic cultures, provided by chlororespiration, which promoted a strong reduction in the PQ pool and a build-up of ΔpH in the thylakoid membranes. These conclusions are consistent with the results related to the performance of PS II under high light. The previous incubation of the cultures under dark anaerobic or aerobic conditions, where a different redox state of PQ was achieved, was reflected in the different levels of induction of xanthophyll cycle pigments. In fact, we observed a rapid and strong induction of photoprotective mechanisms by the induction of the xanthophyll cycle, suggesting a positive contribution of the previous anaerobic over-reduction of the PQ pool to the induction of the xanthophyll cycle, which enhanced the effect of the high light exposure.

The different response of the two cultures was also in agreement with the measurements of oxygen evolution. However, in the anaerobic culture, the re-establishment of a linear electron flow between the two photosystems took place, as shown by the recovery of photosynthetic activity, consistent with previous findings [26,47,48], even if it was always lower than in the aerobic culture and took 1 h to reach the initial level. Furthermore, this difference was due to the extensive reductive conditions of the anaerobic culture, in which the cells maintained a greatly reduced state of the PQ pool during the initial period of exposure to high light.

## 5. Conclusions

According to the previous culture experiments under dark anaerobic conditions, there had already been a decrease in the pH value of the thylakoid lumen in this culture, which was due to its permanence in anaerobiosis. Therefore, exposure to high light maintained a high ΔpH in the thylakoid lumen, which promoted a sustained level of NPQ in the subsequent light phase, which in turn stimulated the xanthophyll cycle. The results demonstrated that anaerobiosis may have a synergistic effect on the induction of zeaxanthin synthesis, with high light intensity, probably due to the previous occurrence of over-reduction in the PQ pool, and hence, when the cells were placed under the high light intensity, the photoprotective mechanism was already alerted to this stress. Therefore, the synergic effect of anaerobic conditions and high light intensity was evidenced.

Our results provide further evidence for the role that a reduction state of the PQ-pool plays in regulating a physiological response of the organism to stress and emphasize the importance of the redox signaling pathway in “alerting” the photosynthetic apparatus to cope with a potential stress situation. These results may help in the implementation of the knowledge of the behavior of photosynthetic microorganisms under stress conditions and increase the possibility of biotechnological applications in large-scale cultivation of microalgae, in outdoor culture, and under sunlight.

## Figures and Tables

**Table 1:** Effects of prolonged dark anaerobiosis on the chlorophyll fluorescence transient (OJIP parameters) in *C. reinhardtii*. M_0_ (initial slope at the beginning of variable fluorescence); V_J_ (variable fluorescence at J; Ψ_0_ (probability that a trapped photon moves an electron beyond Q_A_^−^); ΦE_0_ (probability that an absorbed photon moves an electron beyond Q_A_). For each value, in each condition, different letters indicate significant differences among the values.

Time (min)	M_0_	V_J_	Ψo	ΦEo
0	0.395 ± 0.006 a	0.259 ± 0.005 a	0.742 ± 0.001 a	0.583 ± 0.004 a
240 dark aerobiosis	0.403 ± 0.008 a	0.259 ± 0.008 a	0.748 ± 0.006 a	0.577 ± 0.005 a
240 dark anaerobiosis	1.480 ± 0.132 b	0.651 ± 0.097 b	0.349 ± 0.098 b	0.093 ± 0.011 b

**Table 2:** Induction of xanthophyll cycle in air-bubbled culture (values in the columns “Air”) and nitrogen-bubbled culture (values in the columns “N_2_”), i.e., under aerobic and anaerobic conditions, respectively; D: dark incubation. L: light exposure. ^1^: (V + A + Z): xanthophyll pool; ^2^: DSE = de-epoxidation state (Z + 0.5A)/(V + A + Z); V = violaxanthin, A = antheraxanthin, Z = zeaxanthin, reported as mmol mol^−1^ Chla); Chl: total chlorophyll content (Chl_a+b_). At each time, for each condition, different letters indicate significant differences between the values.

Xanthophyll	Condition	0 min (D)	240 min (D)	15 min (L)	30 min (L)	60 min (L)
**V (mmol mol^−1^ Chl*a*)**	**Air**	104.19 ± 5.30 a	106.76 ± 4.80 a	103.01 ± 4.85 a	94.34 ± 4.72 b	93.19 ± 2.98 b
	**N_2_**	104.19 ± 5.30 a	91.20 ± 4.56 a	78.12 ± 3.28 c	61.55 ± 3.10 d	42.54 ± 1.62 e
**A (mmol mol^−1^ Chl*a*)**	**Air**	2.39 ± 0.07 a	3.23 ± 0.06 a	7.35 ± 0.34 b	9.50 ± 0.47 c	11.05 ± 0.53 d
	**N_2_**	2.39 ± 0.07 a	6.50 ± 0.17 b	13.43 ± 0.63 e	13.95 ± 0.70 e	20.60 ± 1.03 f
**Z (mmol mol^−1^ Chl*a*)**	**Air**	2.46 ± 0.05 a	2.61 ± 0.05 a	5.11 ± 0.12 b	5.40 ± 0.21 b	11.98 ± 0.60 c
	**N_2_**	2.46 ± 0.05 a	3.43 ± 0.17 ab	8.54 ± 0.43 d	21.51 ± 1.01 e	45.52 ± 2.28 f
**(V + A + Z) ^1^**	**Air**	109.04 ± 5.42 a	112.60 ± 4.91 ab	115.46 ± 5.31 b	109.24 ± 5.46 abc	116.22 ± 4.11 c
	**N_2_**	109.04 ± 4.90 a	101.12 ± 4.34 a	100.08 ± 4.81 a	97.00 ± 4.93 a	108.65 ± 4.94 a
**DSE ^2^**	**Air**	0.034 ± 0.001 a	0.038 ± 0.001 b	0.076 ± 0.001 c	0.093 ± 0.000 d	0.151 ± 0.002 e
	**N_2_**	0.034 ± 0.001 a	0.066 ± 0.001 f	0.152 ± 0.001 e	0.283 ± 0.000 g	0.514 ± 0.002 h
**Chl (mg L^−1^)**	**Air**	13.00 ± 0.39 a	12.77 ± 0.22 a	12.50 ± 0.45 a	12.34 ± 0.30 a	13.20 ± 0.11 ad
	**N_2_**	13.00 ± 0.39 a	12.80 ± 0.51 a	12.05 ± 0.57 abc	12.00 ± 0.58 b	13.21 ± 0.40 d

## Data Availability

The original contributions presented in the study are included in the article, further inquiries can be directed to the corresponding author.
